# Risk factors and prevalence of diabetic retinopathy: A protocol for meta-analysis

**DOI:** 10.1097/MD.0000000000022695

**Published:** 2020-10-16

**Authors:** Yuying Hou, Yitong Cai, Zhumin Jia, Suling Shi

**Affiliations:** a The First Affiliated Hospital of Henan University of Science and Technology, Luoyang, Henan; b The school of Nursing, Lanzhou University, Lanzhou, Gansu, China.

**Keywords:** diabetic retinopathy, meta- analysis, risk factors

## Abstract

**Background:**

Diabetic retinopathy (DR) is one of the serious complications of diabetes mellitus. Without further treatment, it can evolve into the stage of proliferation, which will lead to the formation of new blood vessels, vitreous hemorrhage, or anterior retinal hemorrhage, which will lead to severe vision loss and increase the risk of blindness.

**Methods:**

The research literature on the risk factors of diabetic retinopathy published as of July 1, 2020 was searched through MEDLINE, Embase, ovid, Web of Science, Wanfang, CNKI, and other databases, The search strategy has been first developed in MEDLINE using MeSH subject headings combined with free-text terms and Stata12.0 software was used for meta-analysis.

**Results:**

This study is ongoing and the results will be submitted to a peer-reviewed journal for publication.

**Ethics and dissemination:**

Ethical approval is not applicable, since this is an overview based on published articles.

**Protocol registration number:**

The registration number is INPLASY202070107, the DOI number is 10.37766/inplasy2020.7.0107.

## 1 Introduction

Diabetes mellitus (DM) is a group of chronic metabolic diseases caused by genetic, environmental, and autoimmune diseases. Long-term metabolic disorders can lead to microvascular and macrovascular diseases, neurological complications, among others.^[1]^ With the substantial improvement of people's living standards, changes in eating habits and lifestyles, the number of people suffering from diabetes mellitus (DM) is also increasing year by year. According to statistics, there were 425 million people with diabetes in the world aged 20 to 79 years in 2017, and it is expected to increase to 629 million by 2045. Nearly 80% of patients live in low- and middle-income countries (China, India, and so on)^[2]^ According to the WHO study, in 2011, the number of diabetic patients worldwide has reached 366 million, and by 2025, there will be >500 million diabetic patients in the world, and about one-third of them will develop diabetic retinopathy (DR).^[3,4]^

DR is one of the most common and serious microvascular complications in diabetes.^[5]^ Its main pathological changes are the proliferation of capillary endothelial cells, thickening of basement membrane, and selective loss of pericytes, which ultimately leads to the formation of micro angioma, the increase of microvascular permeability, and the blood-retinal barrier destruction, atresia or blockage of capillaries, and formation of new blood vessels.^[6,7]^ At present, the global prevalence rate of DR is 34.6%, and the prevalence rate of DR in developed countries is close to 40.3%^[8]^; 3.6% of patients with type 1 DM and 1.6% of patients with type 2 DM will be blind.^[9]^ DR seriously threatens the quality of life of diabetic patients, and at the same time brings a serious economic burden to society.^[10,11]^

The risk factors of DR are many and complex. It can make the patient progress from asymptomatic step by step and eventually lead to irreversible blindness. However, it is still difficult to effectively prevent the visual impairment caused by it.^[12]^ Therefore, further research on the risk factors of DR and effective preventive measures is necessary. Provide corresponding interventions for high-risk factors to prevent the occurrence and development of DR. The occurrence and development of DR are affected by many factors. Most studies have shown that DR is related to blood sugar level and disease course, but the results of research on the correlation between blood pressure, blood lipids, UAE and body mass index (BMI) and other factors and DR are inconsistent.^[13–15]^

Systematic reviews and meta-analysis can provide scientific evidence for health decisions and can also form higher-level recommendations in the guidelines.^[16–18]^ Adopt the method of meta-analysis of published on angiotensin gene polymorphism, diabetes duration, glycosylated hemoglobin, fasting plasma glucose, postprandial 2 hours blood sugar, triglyceride, cholesterol, uric acid, the relationship between the BMI and DR make a comprehensive analysis of literature, to determine whether the risk factors associated with diabetic retinopathy.

## 2 Methods and analysis

### 2.1 Study registration

This systematic review and meta-analysis has been registered on the International Platform of Registered Systematic Review and Meta-analysis Protocols (INPLASY). The registration number is INPLASY202070107, the DOI number is 10.37766/inplasy2020.7.0107.

### 2.2 Study inclusion and exclusion criteria

#### 2.2.1 2.1 Types of studies

Inclusion: Randomized controlled trial (RCT); cohort studies; case–control studies.

Exclusion: non-Chinese and English literature; incomplete or missing research data; unable to obtain original documents; repeated publication of literature; editorials; commentaries.

#### 2.2.2 Types of participants

DR.

#### 2.2.3 Risk factors

Advanced age, male sex, DM duration, insulin treatment, fasting blood glucose, 2-hour postprandial blood glucose, glycated haemoglobin A1c, total cholesterol, triglyceride, BMI, systolic blood pressure.

#### 2.2.4 Types of outcomes measures

Incidence of DR.

### 2.3 Search scheme and strategy

#### 2.3.1 Electronic searches strategy

“Diabetic retinopathy” was used as the English search term, database retrieval was carried out on MEDLINE, Embase, ovid, Web of Science, Wanfang, CNKI database, and literatures on diabetic retinopathy published from the establishment of the database to July 2019 were collected systematically.

#### 2.3.2 Other resources

Other resources are: manual and other search: search relevant literature by Baidu, Google, Yahoo, and other search engines; document tracing method as an auxiliary retrieval.

### 2.4 Study selection

All search results are imported into EndNote X9 literature management software, 2 reviewers (YYH and ZMJ) will screen the titles and abstracts of literature independently, then read the full text to assess literature according to the inclusion and exclusion criteria, any disagreements will be resolved by a third reviewer (SLS). Study selection will be summarized in a Preferred Reporting Items for Systematic Reviews and Meta-Analyses (PRISMA) flow diagram.

### 2.5 Data extraction

Two researchers (YYH and ZMJ) independently screened the literature in strict accordance with the inclusion and exclusion criteria. During the screening, they first read the title, eliminated the obviously irrelevant literature, and then further read the abstract of the literature and the full text to determine whether to include it or not. If necessary, contact the original study author via email or other means for information. If there is any difference in the content of data extraction, the third party (SLS) shall be consulted.

### 2.6 Risk of bias assessment

Two reviewers will independently assess the quality of included studies by using the Newcastle-Ottawa Scale (NOS) for nonrandomized studies.^[19,20]^ This is a specific method for assessing the quality of cohort and case–control study. There are 8 entries in 3 modules, among which 4 points are selected for study population, 2 points for comparability between groups, and 3 points for measurement of results. The total score ≥6 points is considered as high-quality research literature.

The Cochrane bias risk assessment tool was used to evaluate the final included RCTs: random allocation method; allocation plan concealment; blinding of research subjects and experimenters; blinding of outcome evaluators; completeness of result data; selective reporting of studies Results; other sources of bias, including potential bias related to the specific research design of the study.^[21]^ For each of the above items, make a judgment of “low risk of bias,” “high risk of bias,” and “uncertain risk of bias.” Disagreement will be solved by discussion or by consulting the third person (SLS).

### 2.7 Data synthesis

Statistical analysis was performed on the extracted data using Stata 12.0 software. For measurement data, the weighted mean difference is used as the combined effect size; for binary variable data, the odds ratio (OR) is used as the combined effect size. Use the statistics *I*^2^ and *P* values to test the heterogeneity of the combined literature. If P ≥ .1, *I*^2^ < 50%, it indicates that there is homogeneity among the studies or the heterogeneity is within the acceptable range, and the fixed-effects model is used to merge the calculation of the effect size; on the contrary, it is considered that there is heterogeneity between the studies. Egger method and Begg method were used to assess publication bias.

### 2.8 Subgroup analysis

If the evidence is sufficient, we will conduct a subgroup analysis to determine the difference between different sex, age (>60 years, <60 years) among others.

### 2.9 Quality of evidence

Two reviewers (YYH and ZMJ) will use the GRADE (Grading of Recommendations Assessment, Development and Evaluation) method to assess the quality of evidence of included studies. The evidence levels are classified into 4 levels: high, moderate, low, or very low.

## 3 Discussion

To investigate the risk factors for any DR in people with DM, a random-effects meta-analysis was employed a priori because of anticipated variation in study populations, geography, and study design. As a rule, we only included risk factors that were investigated in at least 3 studies using multivariate design, and the definitions of the same risk factor should be similar across all included studies. Finally, 11 factors met our criteria and were included in meta-analysis.

## Author contributions

**Conceptualization:** Yuying Hou.

**Methodology:** Yitong Cai.

**Software:** Yuying Hou, Zhumin Jia, Suling Shi.

**Writing – original draft:** Yuying Hou, Suling Shi, Zhumin Jia.

**Writing – review & editing:** Yuying Hou, Suling Shi, Zhumin Jia.
